# Subacute invasive pulmonary aspergillosis following breast cancer treatment: A one-year diagnostic odyssey

**DOI:** 10.1016/j.idcr.2025.e02382

**Published:** 2025-09-27

**Authors:** Zahra Sheidae Mehne, Elham Honarjou, Mohammad Reza Sarveghad

**Affiliations:** Department of Infectious Diseases and Tropical Medicine, Faculty of Medicine, Mashhad University of Medical Sciences, Mashhad, Iran

**Keywords:** Subacute invasive pulmonary aspergillosis, Immunocompromised host, Breast cancer, Voriconazole, Diagnostic delay, Molecular diagnostics

## Abstract

**Background:**

Subacute invasive pulmonary aspergillosis (SIPA) presents diagnostic challenges in post-chemotherapy patients, where persistent respiratory symptoms may be attributed to treatment sequelae rather than opportunistic infection. Recognition is often delayed due to non-specific clinical features and presumed immune recovery following cancer treatment completion.

**Case presentation:**

A 39-year-old woman with a history of invasive ductal breast carcinoma developed persistent non-productive cough and intermittent haemoptysis twelve months post-treatment. Despite multiple admissions and empirical therapy over one year, symptoms persisted. Computed tomography (CT) revealed left lower lobe consolidation with cavitation. Diagnosis was established through positive Aspergillus fumigatus PCR and markedly elevated serum Aspergillus IgG (60 NTU; normal <11). Voriconazole treatment achieved clinical improvement and radiological resolution, with 41 % reduction in IgG at four weeks.

**Conclusion:**

This case illustrates the prolonged diagnostic odyssey characteristic of SIPA in cancer survivors and emphasises the critical importance of maintaining clinical suspicion for invasive fungal infections beyond the immediate post-chemotherapy period. Molecular diagnostics combined with Aspergillus-specific serology proved superior to conventional methods, whilst serial antibody monitoring provided valuable biomarker-guided treatment assessment. Early recognition and appropriate antifungal therapy can achieve excellent outcomes even after protracted symptomatic periods.

## Introduction

Subacute invasive pulmonary aspergillosis (SIPA) represents a diagnostically challenging intermediate form of Aspergillus infection, positioned between the rapidly fatal acute invasive disease and the indolent chronic pulmonary forms [1], [2]. This condition predominantly affects patients with mild to moderate immunocompromise, evolving insidiously over weeks to months and frequently masquerading as bacterial pneumonia, tuberculosis, or malignancy recurrence [3]. Unlike the profound neutropenia associated with acute invasive aspergillosis, SIPA typically occurs in patients with subtler immune dysfunction, including those recovering from chemotherapy or receiving chronic immunosuppressive therapy [4].

The post-chemotherapy population presents particular diagnostic complexity, as immune recovery following cancer treatment creates a deceptive sense of restored health whilst persistent vulnerability to opportunistic infections may extend 6–24 months beyond treatment completion [5], [6]. Cancer survivors developing respiratory symptoms often undergo extensive evaluation for malignancy recurrence or treatment-related pulmonary toxicity, whilst invasive fungal infections remain under-recognised despite their significant morbidity potential [7]. The clinical presentation of SIPA—characterised by persistent cough, low-grade fever, haemoptysis, and progressive dyspnoea—lacks pathognomonic features, contributing to diagnostic delays and suboptimal outcomes [8].

Traditional microbiological approaches, including sputum culture and microscopy, frequently yield negative or misleading results in SIPA, necessitating advanced diagnostic strategies [9]. Contemporary management increasingly relies upon molecular diagnostics, particularly Aspergillus polymerase chain reaction (PCR), combined with serological markers such as Aspergillus-specific immunoglobulin G antibodies [10]. Unlike severely immunocompromised patients who may exhibit negative serology, those with SIPA often mount detectable antibody responses, providing both diagnostic utility and valuable biomarkers for treatment monitoring [11].

Early recognition and appropriate antifungal therapy can achieve excellent outcomes, typically involving prolonged azole treatment with voriconazole or itraconazole [8]. However, diagnostic delays remain commonplace, reflecting insufficient clinical awareness and over-reliance on conventional microbiological methods [12]. Serial monitoring of Aspergillus-specific antibodies offers objective assessment of treatment response, though this approach requires further validation in clinical practice [11].

This case report illustrates the protracted diagnostic journey characteristic of SIPA in cancer survivors and demonstrates the clinical utility of comprehensive mycological evaluation incorporating molecular diagnostics and quantitative serology. The case emphasises the critical importance of maintaining clinical suspicion for invasive fungal infections beyond the immediate post-chemotherapy period and provides practical insights into biomarker-guided treatment monitoring strategies.

## Case presentation

A 39-year-old woman with a history of grade II invasive ductal carcinoma of the right breast presented to our institution with chronic cough, intermittent low-grade fever, fatigue, and recent-onset non-massive hemoptysis. Her oncological journey had begun 26 months earlier with hormone receptor-positive, HER2-negative invasive ductal carcinoma, for which she underwent right modified radical mastectomy followed by eight cycles of adjuvant chemotherapy—four cycles of doxorubicin plus cyclophosphamide followed by four cycles of paclitaxel. She subsequently received 23 sessions of radiotherapy to the chest wall and was initially placed on tamoxifen 20 mg daily for hormonal therapy, which was later switched to letrozole 2.5 mg daily due to persistent respiratory symptoms. Genetic testing for BRCA mutations was not performed as she did not meet our institutional criteria, being under 40 years of age without a family history of breast or ovarian cancer and presenting with unilateral disease.

**Fig. 1:**
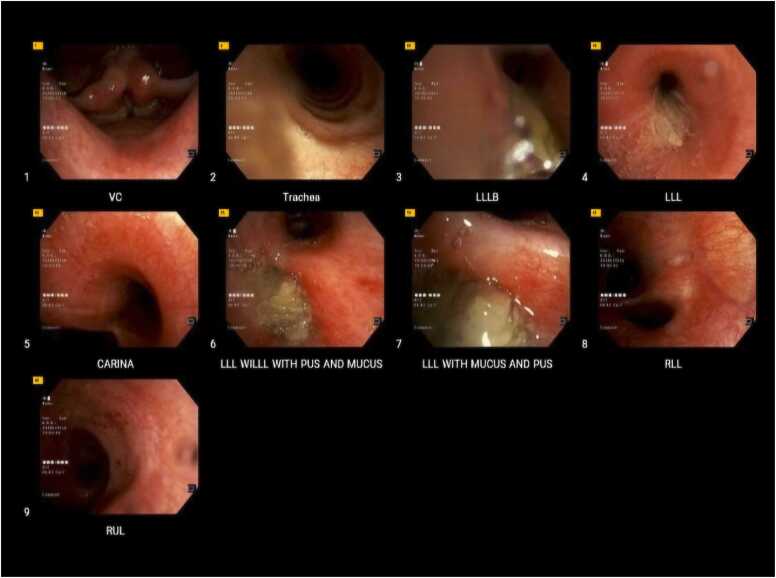
Bronchoscopic findings. Legend: Images demonstrate thick yellow mucoid secretions obstructing the left lower lobe bronchus, consistent with inflammatory exudate secondary to underlying pulmonary aspergillosis.

Over the preceding year, her respiratory symptoms had been persistent and progressively debilitating. She had sought medical attention on multiple occasions and had been treated with several courses of broad-spectrum antibiotics (amoxicillin-clavulanate, azithromycin, levofloxacin) for presumed recurrent bacterial bronchitis or pneumonia, without significant clinical improvement. During one hospitalization approximately nine months after symptom onset, she underwent flexible bronchoscopy (Fig. 1). Bronchial biopsy revealed severe eosinophilic infiltration without evidence of malignancy, while bronchoalveolar lavage culture grew Candida species, prompting treatment with oral fluconazole 200 mg daily for four weeks. This antifungal therapy yielded no improvement in her respiratory symptoms. Comprehensive tuberculosis evaluation including acid-fast bacilli examination and Mycobacterium tuberculosis complex PCR remained consistently negative, as did testing for non-tuberculous mycobacteria.

Her hemoptysis was new in onset during the two months prior to current presentation, described as daily streaks of blood in tenacious sputum. Most recently, she had been admitted to another hospital where, following treatment failure for community-acquired pneumonia, broad-spectrum therapy with intravenous vancomycin and meropenem was administered without clinical improvement, necessitating transfer to our institution for further evaluation. She denied significant weight loss, night sweats, or dyspnea at rest, though her cough was often exacerbated by physical exertion. Her past medical history was otherwise unremarkable, with no known chronic lung disease, diabetes mellitus, or other significant immunosuppressive conditions beyond her cancer treatment. She was a lifelong non-smoker with no significant occupational dust exposures or recent travel history.

### Physical examination

Upon current admission, physical examination was largely unremarkable. Vital signs were stable: temperature 37.2°C, heart rate 82 bpm, blood pressure 118/76 mmHg, respiratory rate 16 breaths/min, oxygen saturation 98 % on room air. Pulmonary auscultation revealed decreased breath sounds at the left lung base, but no adventitious sounds such as rales, rhonchi, or wheezes were appreciated. Cardiovascular and abdominal examinations were normal. There was no evidence of active bleeding or lymphadenopathy.

### Laboratory findings

Initial laboratory workup revealed a normal complete blood count with no leukocytosis, anemia, or thrombocytopenia (WBC 5.2 × 10⁹/L, hemoglobin 15.1 g/dL, platelets 266 × 10⁹/L). Inflammatory markers were mildly elevated (ESR 29 mm/hr, CRP 2.6 mg/L). Comprehensive metabolic panel including renal and hepatic function tests were within normal limits.

### Radiological findings

**Fig. 2:**
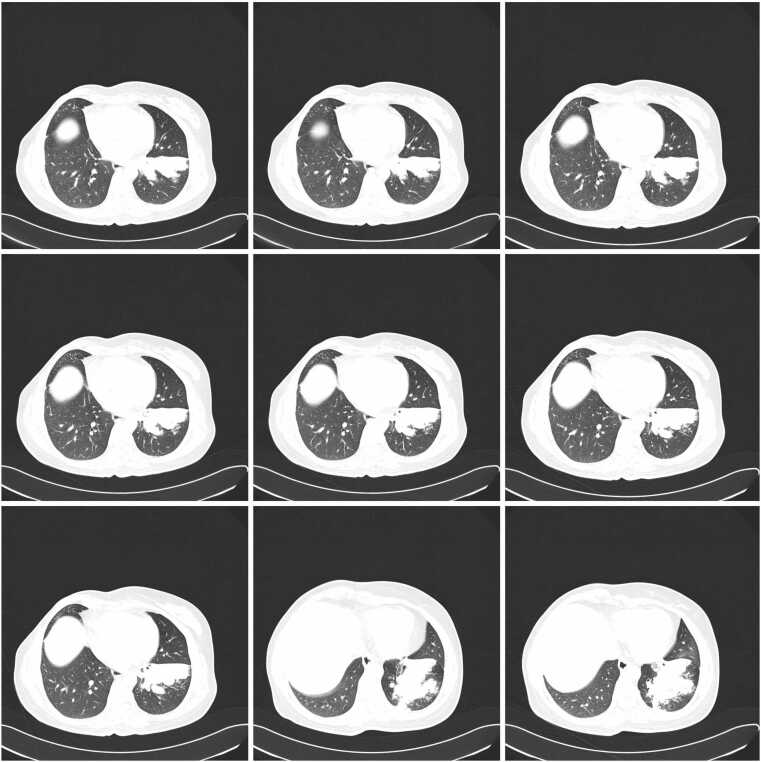

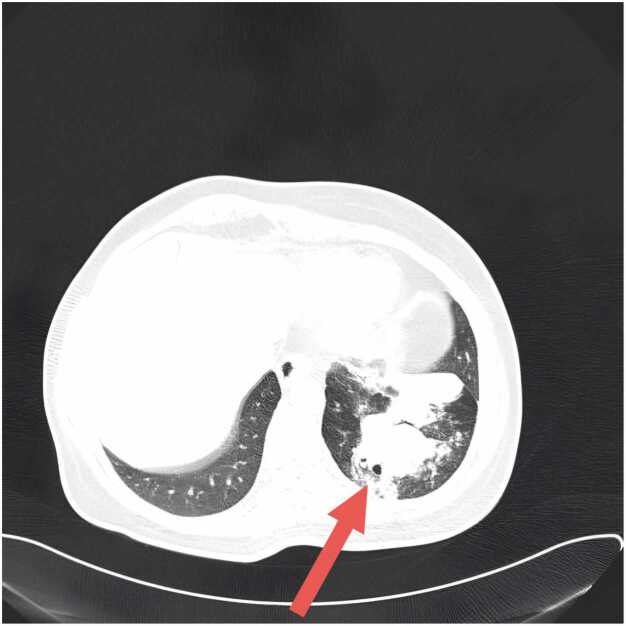
High-resolution computed tomography (HRCT) of the chest demonstrating subacute invasive pulmonary aspergillosis before treatment. A. Serial axial HRCT images (9 consecutive slices) showing a persistent area of consolidation in the left lower lobe with early cavitation. B. Single axial HRCT image demonstrating the maximal extent of the cavitary lesion in the left lower lobe. The arrow indicates areas of central hypodensity within the consolidation, representing liquefactive necrosis progressing to frank cavitation. Air bronchograms are visible within portions of the consolidated lung parenchyma. The lesion is surrounded by a rim of ground-glass opacity, characteristic of the inflammatory halo seen in invasive aspergillosis.

High-resolution computed tomography (HRCT) of the chest revealed a persistent area of consolidation in the superior segment of the left lower lobe, measuring approximately 4 × 3 cm, with several nodular components within it, the largest measuring up to 16 mm in diameter. Notably, there was evidence of central cavitation within the largest nodule, and surrounding ground-glass opacities were present, suggesting adjacent inflammatory changes (Fig. 2). The radiological findings raised concern for atypical infection, possible malignancy recurrence, or metastatic disease given her oncological history. There was no evidence of mediastinal lymphadenopathy, pleural effusion, or contralateral lung involvement. Comparison with previous CT scans from 6 months earlier showed similar but less prominent consolidation without definite cavitation, indicating progressive disease evolution.

### Diagnostic evaluation

Given the lack of response to broad-spectrum antimicrobial therapy, absence of radiological or clinical evidence of malignancy recurrence, and concerning CT findings demonstrating progressive consolidation with central cavitation, subacute invasive pulmonary aspergillosis (SIPA) was considered in the differential diagnosis.

### Mycological investigations

• Sputum Aspergillus fumigatus PCR: A spontaneously expectorated sputum sample was collected and processed for broad-range fungal PCR as well as species-specific Aspergillus detection. The Aspergillus fumigatus PCR returned positive, providing definitive molecular evidence of Aspergillus as the causative pathogen.
• Serum Aspergillus fumigatus IgG: This serological assay was performed given the clinical suspicion of chronic or subacute aspergillosis. The result was markedly elevated at 60 NTU (normal range: <11 NTU), providing strong serological evidence of Aspergillus exposure and active immune response.
• Serum Aspergillus fumigatus IgM: This complementary serological test yielded a result of 13 (positive if ≥11), suggesting recent or ongoing antigenic stimulation and supporting active infection rather than past exposure.
• Serum galactomannan: This biomarker, indicative of Aspergillus cell wall polysaccharide components, measured 0.6 (positive if ≥0.5). While not highly elevated, it exceeded the diagnostic threshold, providing additional supportive evidence for invasive aspergillosis.
• Conventional microbiological studies: Multiple sputum samples were submitted for bacterial culture, mycobacterial culture (including acid-fast bacilli staining and M. tuberculosis PCR), and fungal culture with prolonged incubation. All conventional cultures consistently returned negative for bacterial pathogens, mycobacteria, and fungi after extended observation periods, highlighting the diagnostic limitations of traditional culture-based methods in SIPA.

Based on the cumulative clinical presentation, characteristic radiological findings of progressive consolidation with cavitation, and particularly the positive Aspergillus fumigatus PCR combined with markedly elevated serum Aspergillus IgG antibodies and supportive galactomannan results, a definitive diagnosis of subacute invasive pulmonary aspergillosis was established according to modified EORTC/ECIL criteria.

### Management and follow-up

Following diagnostic confirmation, treatment with oral voriconazole was initiated at a loading dose of 400 mg every 12 h for one day, followed by a maintenance dose of 200 mg orally twice daily. The patient was monitored for clinical response and adverse effects.

The patient showed remarkable clinical improvement within two weeks of starting voriconazole. Her fever resolved completely, and her chronic cough significantly reduced in frequency and severity. Hemoptysis ceased entirely within one week of treatment initiation. By six weeks, she reported near-complete resolution of her respiratory symptoms and significant improvement in her overall well-being and energy levels.

### Serial monitoring and treatment response

• March 1, 2025: Baseline serum Aspergillus IgG = 60 NTU (prior to voriconazole initiation)
• April 5, 2025: After approximately one month of voriconazole therapy, repeat serum Aspergillus IgG = 35.4 NTU, representing a significant decline of over 40 % from baseline, indicating robust immunological response to treatment and pathogen clearance
• Progressive serological improvement: Further decline in Aspergillus IgG titers was noted on subsequent measurements, correlating well with sustained clinical improvement

**Fig. 3:**
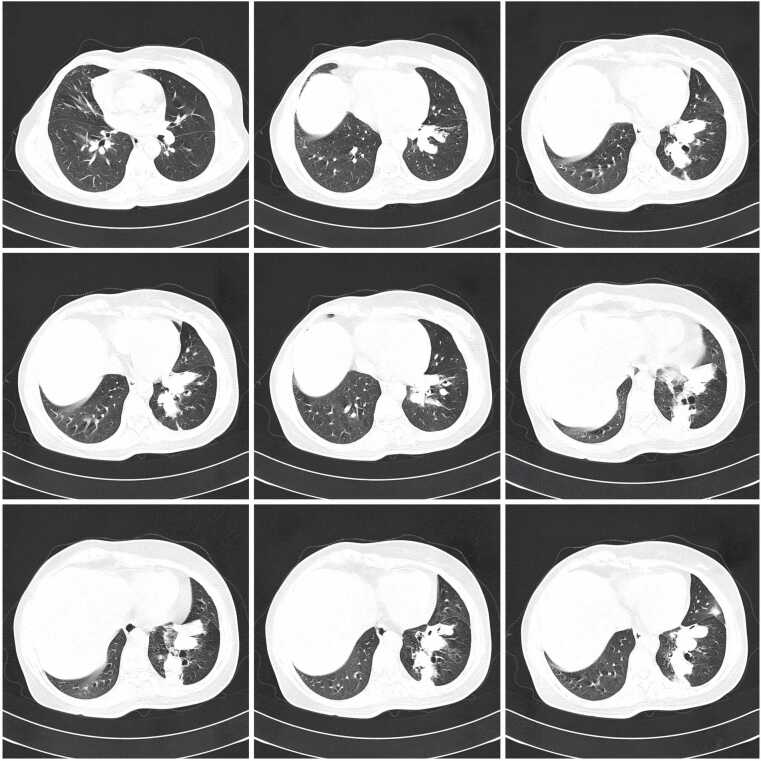

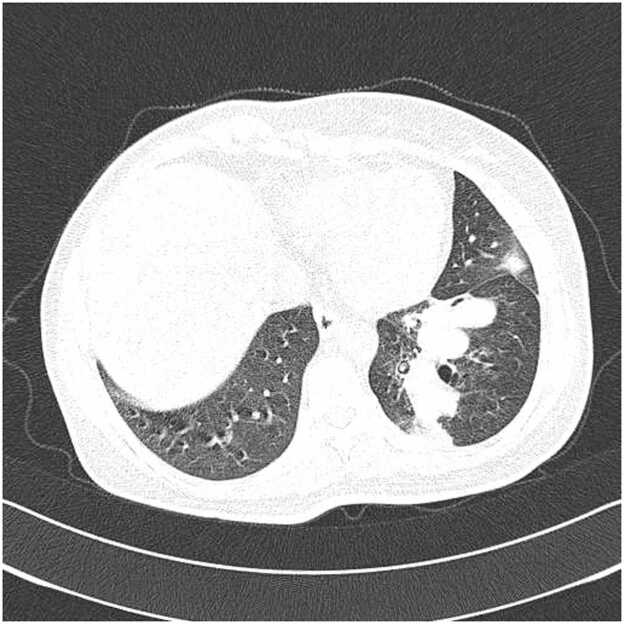
Follow-up HRCT after one month of voriconazole therapy showing treatment response. A. Serial axial HRCT images demonstrating significant improvement with marked reduction in the left lower lobe consolidation. The previously noted cavitary lesion has largely resolved, with only residual thin-walled cystic changes and minimal scarring remaining. The surrounding ground-glass opacities have cleared, indicating resolution of the inflammatory process. These findings confirm excellent response to antifungal therapy. B. Single axial HRCT image at approximately the same level as Fig. 2B, demonstrating significant treatment response after one month of voriconazole therapy. The parenchymal consolidation in the left lower lobe has markedly decreased in size and appears more organized. The previous cavitation has evolved into smaller, more regular cystic or cleft-like changes. The surrounding ground-glass opacity has substantially diminished, indicating resolution of active inflammation. These findings represent excellent therapeutic response to antifungal treatment.

Radiological Follow-up:

Follow-up CT at one month after initiating voriconazole showed significant radiological improvement. The left lower lobe consolidation had markedly diminished, and the central cavitation had resolved, replaced by residual thin-walled cystic changes (Fig. 3). This objective radiological improvement further supported the efficacy of voriconazole therapy.

A planned 6-month course of oral voriconazole was decided upon, considering the subacute nature of the infection, the patient's underlying history of immunosuppression, and the extent of initial disease. Ongoing clinical and laboratory monitoring included regular liver function tests due to potential hepatotoxicity of voriconazole. The patient demonstrated excellent tolerance with no significant adverse effects reported throughout the treatment course.

## Discussion

This case exemplifies the diagnostic challenges inherent in recognising subacute invasive pulmonary aspergillosis amongst post-chemotherapy patients, where persistent respiratory symptoms are frequently attributed to treatment sequelae rather than opportunistic infection. The twelve-month diagnostic odyssey experienced by our patient underscores the critical need for heightened clinical awareness regarding extended immunocompromise following cancer therapy completion.

### Post-chemotherapy vulnerability window

The temporal relationship between our patient’s chemotherapy completion and symptom onset illuminates a crucial yet underappreciated phenomenon. Breast cancer survivors remain susceptible to invasive fungal infections for 6–24 months post-treatment, reflecting persistent T-cell dysfunction and impaired neutrophil function despite apparent clinical recovery [13]. This intermediate immunocompromised state creates optimal conditions for SIPA development, yet recognition remains challenging due to the absence of profound neutropenia typically associated with acute invasive aspergillosis [14].

The pathophysiology of aspergillosis in cancer survivors extends beyond simple neutrophil dysfunction. As comprehensively described by Janssens et al., A. fumigatus accounts for 50–67 % of invasive disease cases, with the clinical syndrome primarily determined by the complex interaction between host immune status and fungal pathogenicity [15]. Their seminal review emphasizes that SIPA represents a distinct clinical entity within the aspergillosis spectrum, characterized by subacute progression over weeks to months in patients with moderate rather than severe immunocompromise. This intermediate phenotype manifests with non-specific symptoms including persistent cough, low-grade fever, and progressive cavitary lung disease, making differentiation from bacterial pneumonia, tuberculosis, or malignancy recurrence particularly challenging [15].

### Diagnostic algorithm optimization and missed opportunities

The twelve-month diagnostic journey in our case warrants detailed analysis. Initial evaluation at another institution focused primarily on malignancy recurrence and community-acquired pneumonia, reflecting common clinical biases in cancer survivors. Despite isolation of Candida species from bronchoalveolar lavage, Aspergillus-specific testing was not performed—a critical missed opportunity. This oversight reflects the persistent misconception that invasive aspergillosis requires profound neutropenia or recent chemotherapy.

The empirical fluconazole treatment for presumed Candida pneumonia represents a diagnostic pitfall, as respiratory Candida isolation typically indicates oropharyngeal contamination rather than true infection in non-neutropenic hosts. When broad-spectrum antibiotics (vancomycin and meropenem) failed to improve symptoms, the diagnostic approach should have expanded to include fungal etiologies, particularly given the cavitary radiological pattern.

Our successful diagnosis relied on molecular methods and serology rather than conventional culture. The positive Aspergillus fumigatus PCR provided definitive species identification where multiple cultures had failed, demonstrating the superior sensitivity of nucleic acid amplification testing in SIPA [16]. The markedly elevated serum Aspergillus IgG (60 NTU) combined with positive IgM indicated active infection with robust antibody response—findings that contrast with severely immunocompromised patients who may exhibit negative serology. The modest galactomannan elevation (0.6) reflects the lower fungal burden in SIPA compared to acute invasive disease, emphasizing that borderline results should not exclude diagnosis when clinical suspicion remains high.

### Biomarker-guided treatment monitoring

**Fig. 4:**
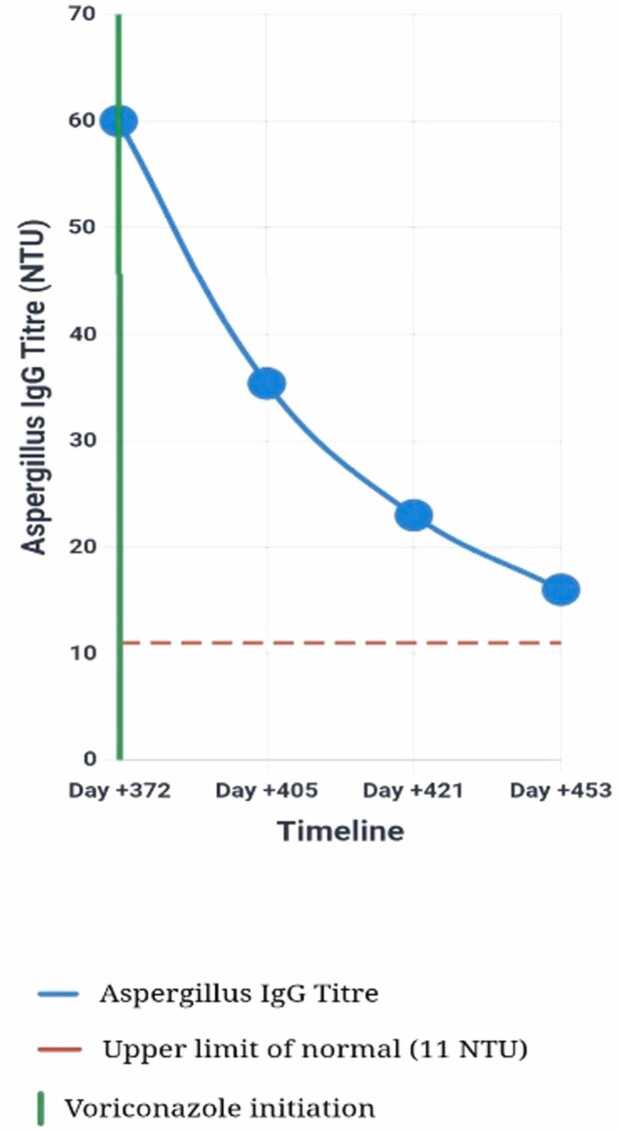
Timeline of Aspergillus IgG titres during therapy. Legend: This line graph illustrates the trend of serum Aspergillus IgG titres (NTU) over time, before and during voriconazole therapy. The red dashed line indicates the upper limit of the normal range for Aspergillus IgG (<11 NTU). The green vertical line marks voriconazole initiation at Day + 372. The initial high titre of 60 NTU (baseline, Day +372) progressively declined to 35.4 NTU at Day + 405, demonstrating a significant reduction (41 %) in response to therapy. Subsequent measurements at Day + 421 and Day + 453 show a continued downward trend to 23 NTU and 16 NTU respectively, correlating with the patient’s clinical and radiological improvement. This serological response pattern supports the efficacy of antifungal therapy in subacute invasive pulmonary aspergillosis.

Serial Aspergillus IgG monitoring provided objective evidence of treatment response, with a 41 % reduction within four weeks correlating with clinical improvement (Fig. 4). This quantitative approach offers advantages over conventional inflammatory markers in assessing antifungal efficacy [17]. The progressive serological decline validated the utility of Aspergillus-specific antibodies as treatment biomarkers in non-neutropenic hosts, supporting recent evidence for their role in chronic aspergillosis management [18].

An important consideration was the potential drug interaction between voriconazole and tamoxifen. Voriconazole inhibits CYP2D6, impairing tamoxifen conversion to its active metabolite, endoxifen. The early switch to letrozole, while initially prompted by respiratory concerns, fortuitously avoided this significant interaction.

### Comparison with reported cases and clinical implications

Our case shares significant clinical parallels with recently published reports of SIPA developing in cancer survivors and other moderately immunocompromised patients [10], [13]. Watanabe et al. described SIPA in a 69-year-old male following chemoradiotherapy for lung cancer, noting the diagnostic challenge in distinguishing fungal infection from malignancy recurrence and radiation-related changes [10]. Similar to our patient, their case demonstrated the insidious nature of SIPA in post-chemotherapy patients, though occurring at the primary treatment site rather than a distant location. Notably, their patient required combination antifungal therapy (liposomal amphotericin B plus voriconazole) due to treatment failure with single-agent micafungin, contrasting with our successful monotherapy approach.

The diagnostic complexity extends beyond cancer survivors, as demonstrated by recent reports of SIPA mimicking tuberculosis both clinically and radiologically across different patient populations. A young diabetic patient presented with similar cavitary lung disease requiring surgical intervention when medical therapy proved insufficient, highlighting the universal challenge of recognising SIPA regardless of underlying risk factors [19]. Dandachi et al. provided comprehensive analysis of invasive pulmonary aspergillosis in solid tumour patients, identifying key risk factors and clinical predictors that align closely with our patient’s presentation, including the post-chemotherapy vulnerability window and delayed diagnostic recognition [13].

Historical perspectives from Pai et al. demonstrated the consistent challenges in diagnosing invasive cavitary pulmonary aspergillosis in cancer patients, emphasising that diagnostic difficulties have persisted across decades despite advances in mycological techniques [13]. Their clinicopathologic study reinforced the importance of maintaining high clinical suspicion in immunocompromised hosts with persistent respiratory symptoms.

Key distinctions in our case include the extended one-year symptomatic period, which likely reflects substantial diagnostic delay rather than the typical 2–3 month course described in classical SIPA [10], [13]. Our utilisation of Aspergillus PCR and serial serum IgG monitoring enabled non-invasive diagnosis and quantitative treatment response assessment—diagnostic strategies not universally available or applied in earlier reports. While other cases relied primarily upon conventional culture methods and imaging [10], [13], molecular and serological approaches offered superior sensitivity in our patient, supporting contemporary recommendations for multi-modality testing in suspected SIPA.

### Radiological correlation and treatment response

High-resolution computed tomography demonstrated marked radiological improvement following voriconazole therapy, with resolution of consolidation and cavitation (Fig. 2, Fig. 3). The initial left lower lobe consolidation measuring 4 × 3 cm with central cavitation progressed to thin-walled cystic changes by one month of treatment, consistent with characteristic imaging evolution in invasive aspergillosis following effective antifungal therapy. This radiological response pattern supports the diagnostic accuracy whilst demonstrating the value of systematic imaging follow-up in treatment monitoring, aligning with established evidence for voriconazole efficacy in achieving both clinical and radiological improvement in invasive pulmonary aspergillosis [11].

## Conclusion

### Clinical decision-making insights

The delayed recognition of SIPA resulted from several factors: attribution of symptoms to post-treatment effects, over-reliance on negative conventional cultures, and initial misinterpretation of Candida colonisation as pathogenic. The presence of eosinophilic infiltration on bronchoscopy, whilst initially suggesting allergic bronchopulmonary aspergillosis, actually represented tissue response to invasive disease. These findings emphasise the importance of comprehensive fungal evaluation when patients fail to respond to empirical antimicrobial therapy, highlighting the need for systematic diagnostic algorithms in this challenging patient population.

### Treatment response and clinical outcomes

Excellent clinical response to oral voriconazole demonstrates that early recognition and appropriate therapy can achieve optimal outcomes even following protracted symptomatic periods. The rapid resolution of fever and haemoptysis within two weeks, coupled with sustained radiological improvement, confirms that SIPA carries favourable prognosis when appropriately managed. This case supports the effectiveness of azole monotherapy in selected patients, whilst highlighting the importance of biomarker-guided monitoring for optimal treatment duration assessment.

## Clinical recommendations

This case supports implementing systematic Aspergillus screening protocols for post-chemotherapy patients presenting with persistent respiratory symptoms exceeding four weeks duration. The combination of molecular diagnostics with serological testing represents the contemporary standard for SIPA evaluation, whilst serial antibody monitoring provides valuable treatment response assessment in appropriately selected patients. Future research should focus on developing standardised diagnostic algorithms and establishing optimal monitoring strategies for this challenging patient population.

## CRediT authorship contribution statement

**Mohammad Reza Sarveghad:** Supervision, Investigation, Conceptualization. **Elham Honarjou:** Writing – review & editing, Supervision, Investigation. **Zahra Sheidae Mehne:** Writing – original draft, Project administration, Data curation.

## Patient consent

Written informed consent was obtained from the patient for publication of this case report and accompanying images. The patient provided explicit consent for the use of clinical data, radiological images, laboratory results, and bronchoscopic findings for educational and research purposes. Patient confidentiality has been maintained throughout, with all identifying information removed or modified. The study was conducted in accordance with the Declaration of Helsinki and institutional ethical guidelines. The patient retains the right to withdraw consent at any time, though no such request has been made to date.

## Funding

This case report received no specific grant from any funding agency in the public, commercial, or not-for-profit sectors. All clinical care, diagnostic testing, and treatment were provided as part of routine patient management at 10.13039/501100004748Mashhad University of Medical Sciences.

## Declaration of Competing Interest

The authors declare that they have no known competing financial interests or personal relationships that could have appeared to influence the work reported in this paper.

## References

[1] Patterson T.F., Thompson G.R., Denning D.W., Fishman J.A., Hadley S., Herbrecht R. (2016). Practice guidelines for the diagnosis and management of aspergillosis: 2016 update by the infectious diseases society of america. Clin Infect Dis.

[2] Denning D.W., Cadranel J., Beigelman-Aubry C., Ader F., Chakrabarti A., Blot S. (2015). Chronic pulmonary aspergillosis: rationale and clinical guidelines for diagnosis and management. Eur Respir J.

[3] Kosmidis C., Denning D.W. (2015). The clinical spectrum of pulmonary aspergillosis. Thorax.

[4] Bassetti M., Peghin M., Vena A. (2018). Challenges and solution of invasive aspergillosis in Non-neutropenic patients: a review. Infect Dis Ther.

[5] Donnelly J.P., Chen S.C., Kauffman C.A., Steinbach W.J., Baddley J.W., Verweij P.E. (2020). Revision and update of the consensus definitions of invasive fungal disease from the european organization for research and treatment of cancer and the mycoses study group education and research consortium. Clin Infect Dis.

[6] Guinea J., Torres-Narbona M., Gijón P., Muñoz P., Pozo F., Peláez T. (2010). Pulmonary aspergillosis in patients with chronic obstructive pulmonary disease: incidence, risk factors, and outcome. Clin Microbiol Infect.

[7] Hoenigl M., Prattes J., Spiess B., Wagner J., Prueller F., Raggam R.B. (2014). Performance of galactomannan, beta-d-glucan, aspergillus lateral-flow device, conventional culture, and PCR tests with bronchoalveolar lavage fluid for diagnosis of invasive pulmonary aspergillosis. J Clin Microbiol.

[8] Jenks J.D., Hoenigl M. (2018). Treatment of aspergillosis. J Fungi.

[9] Kimura Y., Sasaki Y., Suzuki J., Suzuki J., Igei H., Suzukawa M. (2021). Prognostic factors of chronic pulmonary aspergillosis: a retrospective cohort of 264 patients from Japan. PLoS One.

[10] Watanabe H., Shirai T., Saigusa M., Asada K., Arai K. (2020). Subacute invasive pulmonary aspergillosis after chemoradiotherapy for lung cancer. Respirol Case Rep.

[11] Ullmann A.J., Aguado J.M., Arikan-Akdagli S., Denning D.W., Groll A.H., Lagrou K. (2018). Diagnosis and management of aspergillus diseases: executive summary of the 2017 ESCMID-ECMM-ERS guideline. Clin Microbiol Infect.

[12] Rajpurohit R., Wagh P., Heda M., Dubey G., Gujar P.S. (2023). Prevalence of chronic pulmonary aspergillosis in fibrocavitary pulmonary tuberculosis patients. J Fam Med Prim Care.

[13] Dandachi D., Wilson Dib R., Fernández-Cruz A., Jiang Y., Chaftari A.M., Hachem R. (2018). Invasive pulmonary aspergillosis in patients with solid tumours: risk factors and predictors of clinical outcomes. Ann Med.

[14] Gutiérrez-Villanueva A., Diego-Yagüe I., Gutiérrez-Martín I., García-Prieto S., Gutiérrez-Abreu E., Fernández-Guitián R. (2025). Is neutropenia still the main risk factor for invasive aspergillosis? A contemporary university hospital retrospective cohort of invasive aspergillosis in neutropenic and non-neutropenic patients. Ann Clin Microbiol Antimicrob.

[15] Janssens I., Lambrecht B.N., Van Braeckel E. (2024). Aspergillus and the lung. Semin Respir Crit Care Med.

[16] White P.L., Barnes R.A., Springer J., Klingspor L., Cuenca-Estrella M., Morton C.O. (2015). Clinical performance of aspergillus PCR for testing serum and plasma: a study by the european aspergillus PCR initiative. J Clin Microbiol.

[17] Li H., Rui Y., Zhou W., Liu L., He B., Shi Y. (2019). Role of the Aspergillus-Specific IgG and IgM test in the diagnosis and Follow-Up of chronic pulmonary aspergillosis. Front Microbiol.

[18] Page I.D., Richardson M., Denning D.W. (2015). Antibody testing in aspergillosis--quo vadis?. Med Mycol.

[19] akhil S.J.J., Sumesh Raj (2025). Diagnostic dilemma: subacute invasive pulmonary aspergillosis (SAIA) mimicking tuberculosis in a young diabetic patient. Acta Sci Clin Case Rep.

